# Role of *Ascophyllum nodosum* and *Fucus vesiculosus* in Improving the Stress Resistance of *Lactiplantibacillus plantarum*

**DOI:** 10.3390/md23100373

**Published:** 2025-09-25

**Authors:** Sara Frazzini, Matteo Dell’Anno, Luciana Rossi

**Affiliations:** Department of Veterinary Medicine and Animal Sciences—DIVAS, University of Milan, via dell’Università 6, 26900 Lodi, Italy; matteo.dellanno@unimi.it (M.D.); luciana.rossi@unimi.it (L.R.)

**Keywords:** *Lactiplantibacillus plantarum*, *Ascophyllum nodosum*, *Fucus vesiculosus*, prebiotics, probiotic stress tolerance, pH and enzyme resistance, functional food

## Abstract

The survival and efficacy of probiotic bacteria depend on their ability to grow under optimal conditions and withstand environmental stresses, and marine macroalgae are emerging as promising prebiotic sources that may enhance their viability. In this study the effect of *Ascophyllum nodosum* and *Fucus vesiculosus* supplementation (0.5% *w*/*v*) on *Lactiplantibacillus plantarum* was evaluated by assessing growth performance and stress resistance under different conditions, including temperatures (15, 25, 30, 37, 42 °C), pH levels (2.5, 3.5, 4.5, 6.5, 8.5), and enzymatic challenges (pepsin, trypsin, bile). Both algal supplements promoted faster initial growth between 25 and 42 °C, particularly at 37 °C, by reducing the lag phase and increasing the growth rate. Under acidic stress, survival exceeded 80% after 3 h, with significant improvements at pH 2.5 and 3.5 in the presence of seaweeds, while enzymatic assays demonstrated enhanced tolerance against pepsin, trypsin, and bile salts. Overall, supplementation with brown algal biomass provided modest but consistent benefits to *L. plantarum* growth and stress resistance, supporting the use of whole macroalgae as functional additives in probiotic formulations for both human and animal nutrition.

## 1. Introduction

Probiotics are defined as “live microorganisms that, when administered in adequate amounts, confer a health benefit on the host” [1]. They have attracted substantial attention in both human and animal nutrition for their ability to beneficially modulate gut microbiota composition, enhance immune function, improve nutrient utilization, and mitigate gastrointestinal disturbances [2,3]. Among the most studied probiotic species, *Lactiplantibacillus plantarum* is valued for its metabolic versatility, ability to colonize diverse ecological niches, and well-documented health-promoting effects, including antimicrobial activity and modulation of host immune responses [4]. However, the functional benefits of probiotics are closely dependent on their viability and metabolic activity at the site of action [5]. During industrial processing, storage, and gastrointestinal transit, probiotic cells are subjected to environmental stressors such as elevated temperatures, acidic gastric pH, and digestive enzymes, which can significantly reduce cell counts [6]. Strategies to protect probiotics against these stresses are therefore critical to ensure their efficacy in vivo. Marine macroalgae have emerged as promising functional ingredients in this context, owing to their unique biochemical composition. Brown seaweeds such as *Ascophyllum nodosum* and *Fucus vesiculosus* are rich in structural polysaccharides (e.g., alginates, fucoidans, laminarins), phlorotannins, carotenoids, and minerals [7,8]. These bioactive compounds have been associated with antioxidant, antimicrobial, anti-inflammatory, and immunomodulatory properties [9,10]. Notably, *A. nodosum* and *F. vesiculosus* have demonstrated some of the highest total phenolic contents and antioxidant capacities among brown algae, suggesting a strong potential to counteract oxidative stress in biological systems [10]. Beyond their direct bioactivity, macroalgal polysaccharides can function as prebiotic substrates, selectively stimulating the growth and activity of beneficial gut microorganisms [11,12]. The prebiotic potential of *A. nodosum* has been documented in both human and animal models, where its polysaccharide fractions have promoted the growth of lactic acid bacteria, including *L. plantarum* [13], and inhibited the proliferation of enteric pathogens such as *Escherichia coli* [14]. Similarly, *F. vesiculosus* has been shown to exert positive modulatory effects on gut microbiota composition, with recent work suggesting it may be more effective than *A. nodosum* in preventing high-fat-diet-induced dysbiosis [15]. The dual functionality of these macroalgae, as protective agents against environmental stressors and as prebiotic enhancers of probiotic performance, makes them highly relevant for the development of synbiotic approaches. On one hand, their structural and antioxidant components may form a physical barrier around bacterial cells, reducing damage from heat, acidic conditions, and enzymatic degradation [16]. On the other, their fermentable polysaccharides may support probiotic persistence and metabolic activity in the gastrointestinal tract [17]. Despite this promise, studies explicitly investigating the combined protective and prebiotic effects of *A. nodosum* and *F. vesiculosus* on *L. plantarum* under multiple stress conditions remain scarce. Addressing this knowledge gap is particularly relevant in livestock production, where enhancing probiotic stability could improve nutrient utilization, reduce reliance on antibiotics, and support animal welfare [18], as well as in human nutrition, where similar approaches may aid in the prevention and management of gastrointestinal and metabolic disorders. The present study was therefore undertaken to evaluate the ability of *A. nodosum* and *F. vesiculosus* to preserve *L. plantarum* viability when subjected to thermal, acidic, and enzymatic stress, while also assessing their potential prebiotic contribution. By combining stress-tolerance assays with consideration of prebiotic functionality, this work aims to inform the formulation of next-generation functional foods and feeds that effectively integrate macroalgal bioactives with probiotic cultures.

## 2. Results

### 2.1. Temperature Tolerance

**Figure 1:**
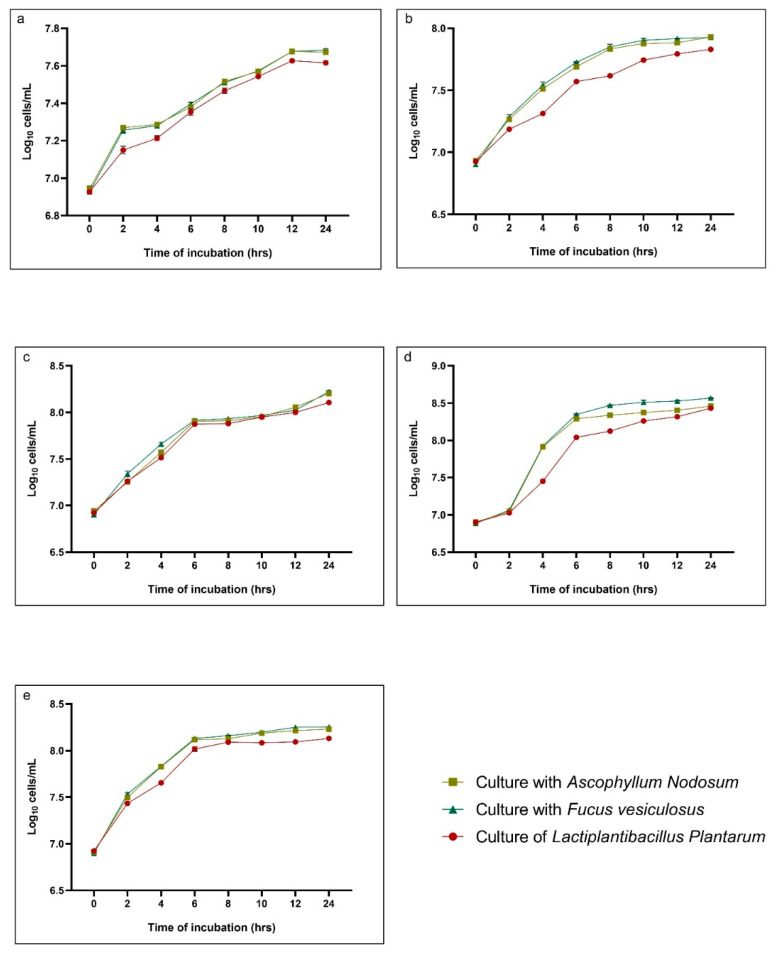
Growth curves of *Lactiplantibacillus plantarum* in pure culture (red line) and in cultures supplemented with *Ascophyllum nodosum* (brown line) or *Fucus vesiculosus* (green line) at 0.5% (*w*/*v*), incubated at (**a**) 15 °C, (**b**) 25 °C, (**c**) 30 °C, (**d**) 37 °C, and (**e**) 42 °C. Bacterial growth was expressed as log_10_ cells/mL over a 24 h incubation period. Values represent the mean ± standard deviation.

The growth performance of *Lactiplantibacillus plantarum* was evaluated at 15, 25, 30, 37, and 42 °C in pure culture and in cultures supplemented with *Ascophyllum nodosum* or *Fucus vesiculosus* (Figure 1). At 15 °C (Figure 1a), all cultures exhibited a slow growth rate, reaching ~7.6 log_10_ cells/mL after 24 h, with no significant differences between treatments across all time points (*p* > 0.05). At 25 °C (Figure 1b), cultures supplemented with algae showed a slightly higher growth than the pure culture, particularly after 4–12 h, with differences between *L. plantarum* and both algal treatments being significant at 6 h and 8 h (*p* < 0.0001). At 30 °C (Figure 1c), all cultures reached ~8.4 log_10_ cells/mL by 24 h, with *F. vesiculosus*-supplemented cultures displaying significantly higher counts than the pure culture at 4 h and 6 h (*p* < 0.0001 and *p* = 0.0481, respectively). At 37 °C (Figure 1d), which supported the highest growth rates, cultures with algae outperformed the pure *L. plantarum*, showing significant differences as early as 4 h (*p* < 0.0001 for both algae vs. pure culture) and maintaining this advantage up to 12 h. Notably, *A. nodosum* and *F. vesiculosus* did not significantly differ from each other at any time point (*p* > 0.05). At 42 °C (Figure 1e), growth was slightly reduced compared to 37 °C, with final counts around 8.3 log_10_ cells/mL. Significant differences were observed between pure cultures and both algal-supplemented cultures at 4 h, 6 h, and 12 h (*p* < 0.0001), while the two algal treatments showed comparable growth throughout the incubation (*p* > 0.05). Overall, supplementation with either *A. nodosum* or *F. vesiculosus,* indicated by the brown line and the green line, respectively, in Figure 1, tended to enhance the growth rate, particularly at optimal and supra-optimal temperatures.

### 2.2. pH Tolerance

The survival rates of *Lactiplantibacillus plantarum* in pure culture and when supplemented with *Ascophyllum nodosum* or *Fucus vesiculosus* at 0.5% (*w*/*v*) under different pH conditions are shown in Table 1. Values were normalized for each time point against the pure culture at pH 6.5 (set to 100%). At 1 h, all cultures maintained high viability across the pH range (97.20–104.76%), with significant differences between treatments at the same pH (*p* < 0.05). Both algal-supplemented cultures showed significantly higher survival than the pure culture at all pH values, with *F. vesiculosus* generally yielding the highest percentages. Differences between pH values within the same treatment indicated that, in the pure culture, survival at pH 4.5, 6.5, and 8.5 was significantly higher than at pH 2.5 and 3.5. After 2 h, survival rates declined, particularly at lower pH values. At pH 2.5, viability dropped to 89.90% in the pure culture but remained significantly higher in the algal-supplemented cultures (93.22–93.48%). The greatest differences between treatments were observed at pH 8.5, where both *A. nodosum* and *F. vesiculosus* maintained survival above 102% compared to 98.72% in the pure culture. At 3 h, further reductions in survival were evident, especially at pH 2.5 (83.37% in the pure culture vs. 85.41–86.12% in supplemented cultures). Across all acidic pH values (2.5–4.5), algal supplementation consistently provided a statistically significant survival advantage (*p* < 0.05). In the pure culture, survival at pH 6.5 remained at 100%, while supplemented cultures showed slightly higher values (101.91%). At alkaline pH 8.5, the pure culture retained 96.37% viability, with *A. nodosum* and *F. vesiculosus* maintaining significantly higher survival (98.29% and 97.93%, respectively).

### 2.3. Enzymatic Tolerance Assay

For pepsin (Table 2), at 0.3 mg/mL, the culture of *L. plantarum* showed a gradual but significant decrease in viability over time (from 7.46 log_10_ CFU/mL to 7.32 log_10_ CFU/mL after three hours of incubation; *p* < 0.05), whereas both *A. nodosum* and *F. vesiculosus* supplemented cultures maintained counts close to the initial level, with significantly higher values than the culture of *L. plantarum* alone at all post-inoculation time points (*p* < 0.05). Similar trends were observed at 0.5 mg/mL and 1.0 mg/mL, with the algal-supplemented cultures showing greater stability, particularly *A. nodosum*, which maintained values ≥ 7.50 log_10_ CFU/mL even after three hours of incubation at the highest enzyme concentration.

Under trypsin stress (Table 3), all treatments exhibited some decline over time, but the reduction was generally smaller than with pepsin. At 0.1 mg/mL, *A. nodosum* supplementation consistently yielded significantly higher counts than the pure culture at 1–3 h (*p* < 0.05), while *F. vesiculosus* showed significant differences mainly at later time points. At 0.5 mg/mL, *A. nodosum* maintained a significant advantage throughout the incubation, and at 1.0 mg/mL, both algae resulted in higher counts than the culture of *L. plantarum* at most time points, with *A. nodosum* again showing the highest stability.

Bile salt tolerance assays revealed a concentration-dependent effect (Table 4). At 0.3% (*w*/*v*), the pure culture experienced a marked decrease over time (from 7.73 to 7.55 log_10_ CFU/mL; *p* < 0.05), whereas *A. nodosum*-supplemented cultures maintained significantly higher counts at all time points (*p* < 0.05), and *F. vesiculosus* showed intermediate performance. At 0.5%, the differences between treatments were less consistent, but *A. nodosum* generally retained higher viability than the culture of *L. plantarum* after 2–3 h. At 1%, viability was essentially stable for all treatments, though small but statistically significant advantages of the algal-supplemented cultures were observed at 1–2 h.

## 3. Discussion

The effectiveness of probiotic microorganisms depends not only on their inherent functional properties but also on their ability to proliferate under favorable conditions and to survive environmental stresses encountered during processing, storage, and gastrointestinal transit [3,19]. Prebiotics, non-digestible food components that selectively stimulate the growth and/or activity of beneficial microorganisms, play a pivotal role in enhancing probiotic performance [12]. In both animal and human nutrition, prebiotic supplementation has been linked to improved gut health, better nutrient utilization, and enhanced resistance to enteric pathogens, contributing to overall host well-being and productivity [15,18]. *Lactiplantibacillus plantarum* is a versatile lactic acid bacterium with a broad ecological niche and recognized probiotic potential; however, its growth and stress tolerance can be modulated by environmental parameters and by the presence of specific dietary substrates [20]. Brown seaweeds such as *Ascophyllum nodosum* and *Fucus vesiculosus* are rich in bioactive compounds with demonstrated prebiotic-like effects, capable of stimulating lactic acid bacteria in vitro and in vivo [11,21]. In this study, the growth and survival of *L. plantarum* in pure culture and supplemented with *A. nodosum* or *F. vesiculosus* (0.5% *w*/*v*) were evaluated under varying temperatures, pH levels, and enzymatic challenges to determine whether brown-algal biomass could confer functional advantages relevant to probiotic applications in both animal and human nutrition.

Across the five incubation temperatures tested, *L. plantarum* displayed the expected mesophilic profile, with fastest growth at 37 °C, slightly reduced performance at 42 °C, and slow but measurable growth at 15 °C. These models are consistent with previous reports that identify ideal growth temperatures as between 30 and 37 °C and describe a reduction in growth at temperatures above the optimum [22,23]. The addition of *A. nodosum* or *F. vesiculosus* significantly accelerated the initial growth of the bacterial culture even at temperatures outside the optimal range, resulting in an increase of approximately 0.1–0.3 log_10_ cells/mL compared to the pure culture between 4 and 12 h, particularly at 37 °C. While the absolute difference diminished by 24 h, the algal treatments consistently shortened the apparent lag phase and increased the apparent growth rate. No meaningful difference emerged between the two seaweed species, suggesting that common brown-algal constituents, such as fucoidans, laminarans, and polyphenols, may emphasize the effect. These observations aligned with the growing evidence that brown-seaweed polysaccharides exert prebiotic-like actions on lactic acid bacteria. Reviews and in vitro studies show that seaweed-derived carbohydrates selectively stimulate *Lactobacillus* spp. and other beneficial microbes [24,25]. From a quantitative perspective, it has been demonstrated that fucoidan increases the number of *Lactobacillus reuteri* by approximately 2 log_10_ in 48 h in vitro, a greater effect than the initial advantage of approximately 0.1-0.3 log_10_ observed in the present study, probably reflecting the specific responses of the probiotic species, the use of purified polysaccharides compared to whole powder biomass, and different culture media and endpoints [26]. Beyond single-strain systems, batch-fermentation studies with *A. nodosum* or other brown seaweeds report increases in beneficial bacterial groups and modulation of community composition, supporting a general growth-promoting potential of brown-algal inputs [14,27]. The effects observed could be attributed to the supply of readily fermentable polysaccharides, which provide an immediate energy source and may shorten the lag phase. In fact, as demonstrated by Zhao and colleagues (2024), the specificity of the substrate is a key factor since its composition, availability, and concentration directly influence the microorganisms’ growth [28]. As reported by McGuire et al., (2023) higher concentrations of substrate reduce the dormancy phase and accelerate growth [29].

Moreover, florotannins, polyphenols extracted from brown algae, offer significant protection against heat stress through both direct action and modulation of cellular pathways. Their efficacy is manifested in the enhancement of enzyme activity, membrane integrity, and stress response signaling. Specifically, florotannins act through direct capture of reactive oxygen species (ROS) and lipid protection. Extracts rich in algae polyphenols and florotannins are able to reduce lipid peroxidation, as measured by the lower level of malondialdehyde, and “scavenge” ROS in cells and tissues, preventing oxidative damage [30,31]. Additionally, the presence of algae bioactive molecules could activate the Nrf2 transcription factor pathway and induce the activity of antioxidant enzymes. This activation leads to increased expression of catalase, glutathione-S-transferase (GST), superoxide dismutase (SOD), and glutathione peroxidase (GPx) in cellular systems and in heat-stressed fish [30,32]. When exposed to different pH values (2.5–8.5), *L. plantarum* maintained high survival, generally above 80% after three hours, and supplementation with either alga biomass significantly improved tolerance under both acidic and alkaline conditions, with a higher protective effect observed at pH 2.5 and 3.5. Acid stress typically compromises membrane integrity and enzyme activity in lactic acid bacteria; however, high-molecular-weight algal polysaccharides may form a physical barrier around cells, thereby reducing proton influx and membrane damage [33,34]. Evidence of this was reported in a study conducted by Chakraborty (2015) in which *Lacticaseibacillus acidophilus* cells encapsulated in alginate showed a decrease in viability of only 4-log after exposure to pH 1.2, while unencapsulated cells were destroyed [35]. Also, other studies have confirmed that encapsulation in alginate protects *Lactobacillus plantarum* even in lactic acid environments of up to 2% [36]. Similar trends have been reported for brown-seaweed extracts, Shannon and Abu-Ghannam (2016) [21] showed that *Laminaria digitata*, *Fucus serratus*, and *Ascophyllum nodosum* extracts improved *Lactobacillus rhamnosus* survival at pH 2.0 by up to 2 log_10_ CFU/mL, while Zou et al. (2021) [37] found that *A. nodosum* extract enhanced acid stability of *L. brevis* and *L. casei*, likely through high-molecular-weight polysaccharides forming a protective barrier. Comparative studies on other algal sources have also reported acid-protective effects. Ganchev et al. (2024) [38] observed that *Spirulina platensis* biomass increased the survival of *L. plantarum* by 1.5 log_10_ CFU/mL at pH 2.5, attributing this effect to the combined action of polysaccharides and antioxidant pigments. Wimmer et al. (2021) reported that fucoidan from *Undaria pinnatifida* enhanced the survival of *L. acidophilus* under acidic conditions [39]. In addition to the protective effect of algae biomasses, this study also disclosed that *F. vesiculosus* outperformed *A. nodosum* during the first hour of exposure at low pH, while *A. nodosum* showed more consistent performance over prolonged incubation. Such differences may reflect variations in the composition and solubility of polysaccharides between the two algae species [40].

A similar pattern emerged under enzymatic stress, where the supplementation with *A. nodosum* or *F. vesiculosus* enhanced survival in the presence of pepsin, trypsin, and bile salts, with the largest effects observed against pepsin and low-concentration bile salts. Algal polysaccharides, such as fucoidans and laminarans, have been reported to protect microorganisms from digestive enzymes and bile stress. This multifaceted defense mechanism is based on different pathways; first of all, a direct inhibition of proteolytic enzymes, like pepsin and trypsin, was reported. Quantitative data shows that fucoidan fractions, from *Fucus vesiculosus* and *Undaria pinnatifida*, can reduce enzymatic activity by 25% to 75% [41]. This process relies on a direct binding between the polysaccharides and the enzymes, although the effectiveness varies with specific compositions [42,43]. A second mechanism is the sequestration of bile salts. Negatively charged polysaccharide fractions, particularly those rich in uronic acids, are highly effective at binding and sequestering bile salts, which lowers their concentration and limits potential damage to bacterial cell membranes [44,45]. Finally, the antioxidant activity of algal polysaccharides is well-documented; they help reduce oxidative stress and inflammation by activating signaling pathways like the Nrf2 pathway [46]. The improvement observed was smaller than the effects reported for purified extracts. For instance, Shannon and Abu-Ghannam (2016) [21] documented a ~1.8 log_10_ CFU/mL increase in *L. rhamnosus* survival in simulated gastric juice with pepsin, and Yang et al. (2024) [47] reported a 1.2 log_10_ CFU/mL improvement for *L. acidophilus* under combined acid and bile stress with *Undaria pinnatifida* fucoidan. Venardou et al. (2021) [14] also found that *A. nodosum* extract increased *L. brevis* bile tolerance by 0.9 log_10_ CFU/mL at 0.3%. The stronger effects reported in the literature could be due to higher active compound concentrations in extracts compared to whole biomass. However, comparable benefits have been described for *Spirulina platensis* biomass that increased *L. plantarum* tolerance to acid and bile by up to 1.5 log_10_ CFU/mL [38].

Overall, the results of this study show that brown-algal biomass can provide modest but consistent benefits to *L. plantarum*, accelerating early growth at optimal and moderately stressful temperatures, enhancing survival across a broad pH range, and improving resistance to digestive enzymes. These effects could be valuable for developing more robust probiotic formulations, particularly in functional foods subjected to thermal processing, acidic environments, or gastrointestinal transit. While purified algal extracts may offer greater protective effects, whole biomass represents a cost-effective, minimally processed alternative. The observed benefits are compatible with a multifactorial mechanism involving nutrient supply, antioxidant activity, and physical or chemical protection, which nevertheless warrants further exploration in future studies. In parallel, these mechanistic insights also highlight the practical potential of *A. nodosum* and *F. vesiculosus* as functional ingredients in human foods and animal feeds. In human nutrition, whole seaweed powders or extracts could be incorporated into fermented dairy products, beverages, or dietary supplements, while in animal production, dried algal biomass could be added directly to feed formulations to improve probiotic stability and gut health. However, challenges such as taste or palatability, variability in seaweed composition, and the stability of bioactive compounds during storage and processing must be carefully addressed to translate these benefits into consistent and applicable solutions.

## 4. Materials and Methods

### 4.1. Seaweed Biomass

*Ascophyllum nodosum* was purchased from Italfeed Srl (Milan, Italy), while *Fucus vesiculosus* was purchased from Sevecom S.p.a. (Milan, Italy) both in confirmation with European safety requirements.

### 4.2. Bacterial Strain and Culture Condition

The algae concentration used in the study was 0.5% (*w*/*v*), selected according to previous experiments from our research group, where different inclusion levels were tested and 0.5% (*w*/*v*) ensured the most consistent and linear growth of *L. plantarum* strain [13]. *Lactiplantibacillus plantarum* strain derived from our laboratory stock at −80 °C [48] into the De Man, Rogosa and Sharpe (MRS) medium (Sigma-Aldrich, St. Louis, MO, USA) and incubated at 35 ◦C for 24 h under a microaerophilic atmosphere.

### 4.3. Survival of Lactiplantibacillus plantarum Cultured in the Presence of Seaweed Biomass Under Adverse Environmental Conditions

#### 4.3.1. Temperature Tolerance Assay

The effect of incubation temperature on the growth of *Lactiplantibacillus plantarum* was evaluated in pure culture and in the presence of algal biomass (*A. nodosum* and *F. vesiculosus*) following procedures adapted from Gökmen et al. (2024), with minor modifications [23]. The strain was cultured in MRS broth (Sigma-Aldrich, St. Louis, MO, USA) and incubated at 37 °C for 18 h to obtain an overnight culture in the late exponential/early stationary phase. Cells were harvested by centrifugation at 5000× *g* for 10 min at 4 °C, washed twice with sterile phosphate-buffered saline (PBS, pH 7.2), and resuspended in fresh MRS broth to obtain an initial optical density at 600 nm (OD_600_) of approximately 0.1 (≈10^7^ CFU/mL). The same procedure was followed for the algae-supplemented treatments, where *A. nodosum* and *F. vesiculosus* dried powders were added to MRS broth (0.5%, *w*/*v*), immediately before inoculation. Aliquots of the standardized bacterial suspensions, with or without algal biomass, were distributed into sterile culture tubes and incubated statically at 15 °C, 25 °C, 30 °C, 37 °C, and 42 °C. Growth was monitored by measuring the optical density (OD_600_) at two-hour intervals for a total period of 24 h using a spectrophotometer (BioTek Epoch, Agilent, Santa Clara, CA, USA). For each temperature and treatment, three independent biological replicates were performed.

#### 4.3.2. pH Tolerance Assay

The tolerance of *Lactiplantibacillus plantarum* to different pH values was evaluated in both pure cultures and in cultures supplemented with algal biomass, following methodologies adapted from Charteris et al. (1998) [33]. An overnight culture of *L. plantarum* was grown in MRS broth (Sigma-Aldrich, St. Louis, MO, USA) at 37 °C for 18 h. Cells were harvested by centrifugation at 5000× *g* for 10 min at 4 °C, washed twice with sterile phosphate-buffered saline (PBS, pH 7.2), and resuspended in fresh MRS broth, with or without algal biomass, to obtain an initial cell density of approximately 10^8^ CFU/mL. For the assay, MRS broth was adjusted to the target pH values of 2.5, 3.5, 4.5, 6.5, and 8.5 using sterile 1 M HCl or 1 M NaOH. Aliquots of pH-adjusted broth were inoculated with the standardized bacterial suspension and incubated statically at 37 °C. Cell viability was assessed after 1 h, 2 h, and 3 h of incubation at each pH by withdrawing 1 mL samples, performing ten-fold serial dilutions in sterile PBS, and plating in duplicate using the pour plate method with MRS agar. Plates were incubated at 37 °C for 48 h, and colonies were counted to determine viable cell counts. Survival rates were expressed as the percentage of viable cells compared to the control, represented by the culture of *L. plantarum* alone, at pH 6.5 at the same time point.

#### 4.3.3. Enzyme Tolerance Assay

The tolerance of *Lactiplantibacillus plantarum* to gastrointestinal enzymes was evaluated in both pure cultures and in cultures supplemented with algal biomass, following methodologies adapted from Oguntoyinbo et al. (2015) and Singhal et al. (2021), with modifications [49,50]. Test cultures were prepared as described in Section 4.3.2.

To determine the pepsin tolerance, bacterial suspensions were inoculated into 9 mL of sterile glycine–HCl buffer (pH 2.5) containing pepsin (porcine gastric mucosa, ≥2500 U/mg; Sigma-Aldrich, St. Louis, MO, USA) at final concentrations of 0.3, 0.5, and 1.0 mg/mL, as reported in Kouadio et al. (2024) [51]. Tubes were incubated statically at 37 °C, and samples were collected at 1, 2, and 3 h. Pepsin activity was neutralized by adjusting the pH to ~6.5–7.0 with sterile NaOH (1 M) before enumeration.

For trypsin tolerance, suspensions were inoculated into sterile Tris–HCl buffer (pH 7.5) containing trypsin (porcine pancreas, ≥8000 U/mg; Sigma-Aldrich, St. Louis, MO, USA) at final concentrations of 0.1, 0.5, and 1.0 mg/mL [49]. Samples were taken at the same time points as above. Trypsin was inactivated by centrifugation (5000× *g*, 10 min) and resuspension in sterile PBS prior to plating.

Finally, the bile tolerance was assessed by inoculating the bacterial suspension into sterile PBS (pH 7.0) containing bile salts (Sigma-Aldrich, St. Louis, MO, USA) at final concentrations of 0.3, 0.5, and 1.0% (*w*/*v*) [50]. Samples were collected at the same time points as the other assays, centrifuged (5000× *g*, 10 min), and resuspended in PBS before enumeration.

For all treatments, 1 mL samples collected at each time point were serially diluted in sterile PBS and plated in duplicate using the pour plate method with MRS agar. Plates were incubated at 37 °C for 48 h, and colonies were counted to determine viable cell counts, expressed as log_10_(CFU/mL).

### 4.4. Statistical Analysis

All experiments were performed in biological duplicate and technical triplicate using independent bacterial cultures. Bacterial counts were expressed as log_10_ CFU/mL before statistical analysis to normalize variance and approximate a Gaussian distribution. For pH tolerance assays, survival rates were calculated by normalizing the viable counts at each pH value to the corresponding counts obtained for the *L. plantarum* control at pH 6.5 at the same incubation time point. Growth curves under different temperature conditions were analyzed by two-way repeated measures analysis of variance (ANOVA) with treatment and time as fixed factors. Separate two-way ANOVAs were performed for the pH and enzyme tolerance assays, with treatment and pH level or enzyme concentration as the fixed factors, and time as an additional repeated measure when applicable. Interaction terms between factors were included in all models to assess whether the effect of treatment varied according to environmental condition or exposure time. When significant effects were detected, Tukey’s honestly significant difference (HSD) post hoc test was applied for pairwise comparisons. All statistical analyses were performed using GraphPad Prism version 9.0 (GraphPad Software, San Diego, CA, USA), with significance set at *p* < 0.05.

## 5. Conclusions

The present study demonstrated that supplementation of *Lactiplantibacillus plantarum* with *Ascophyllum nodosum* or *Fucus vesiculosus* could confer measurable functional advantages under a range of environmental stresses relevant to probiotic survival. Both seaweed species modestly enhanced early growth rates at optimal and moderately stressful temperatures, increased tolerance across a wide pH range, particularly under strong acid stress, and improved survival in the presence of digestive enzymes, with the most pronounced effects observed against pepsin and low bile salt concentrations. While the observed benefits were generally modest compared to those reported for purified algal extracts, they were consistent across different stress conditions and did not differ substantially between the two seaweed species. These findings suggest that whole brown-algal biomass, as an affordable and minimally processed ingredient, has potential as a functional additive in probiotic formulations for both human and animal nutrition. Future research should focus on elucidating the mechanisms underlying these effects, exploring dose–response relationships, and validating the findings under in vivo conditions.

## Figures and Tables

**Table 1:** Survival rates (%, mean ± standard deviation) of *Lactiplantibacillus plantarum* in pure culture and supplemented with *Ascophyllum nodosum* or *Fucus vesiculosus* (0.5% *w*/*v*) at different pH values (2.5, 3.5, 4.5, 6.5, and 8.5) after 1, 2, and 3 h of incubation. Values were normalized at each time point to the corresponding pure culture at pH 6.5 (set as 100%). ^a,b,c^ Different lowercase letters within the same row indicate significant differences (*p* < 0.05) between treatments at the same pH. ^A,B,C,D,E^ Different uppercase letters within the same column indicate significant differences (*p* < 0.05) between pH values within the same treatment.

		Survival Rate (%)		
Time	pH	*L. plantarum*	*L. plantarum* with *A. nodosum*	*L. plantarum* with *F. vesiculosus*
1 h	2.5	97.20 ± 0.133 ^a, B^	99.07 ± 0.133 ^b, B^	99.29 ± 0.077 ^b, B^
	3.5	98.40 ± 0.133 ^a, C^	99.29 ± 0.077 ^b, B^	99.47 ± 0.133 ^b, B^
	4.5	100.18 ± 0.154 ^a, A^	100.84 ± 0.154 ^b, C^	103.11 ± 0.077 ^c, C^
	6.5	100.04 ± 0.539 ^a, A^	101.78 ± 0.770 ^b, A^	104.76 ± 0.154 ^c, A^
	8.5	99.60 ± 0.231 ^a, A^	101.47 ± 0.231 ^b, AC^	103.20 ± 0.533 ^c, C^
2 h	2.5	89.90 ± 0.221 ^a, B^	93.22 ± 0.128 ^b, B^	93.48 ± 0.128 ^b, B^
	3.5	93.22 ± 0.128 ^a, C^	94.63 ± 0.128 ^b, C^	94.76 ± 0.126 ^b, C^
	4.5	97.66 ± 0.450 ^a, D^	98.59 ± 0.128 ^b, D^	101.41 ± 0.130 ^c, D^
	6.5	100.00 ± 0.256 ^a, A^	102.98 ± 0.195 ^b, A^	103.41 ± 0.074 ^b, A^
	8.5	98.72 ± 0.256 ^a, E^	102.47 ± 0.391 ^b, A^	102.13 ± 0.195 ^b, E^
3 h	2.5	83.37 ± 0.120 ^a, B^	85.41 ± 0.120 ^b, B^	86.12 ± 0.120 ^c, B^
	3.5	85.29 ± 0.207 ^a, C^	86.60 ± 0.120 ^b, C^	87.32 ± 0.120 ^c, C^
	4.5	92.46 ± 0.120 ^a, D^	94.30 ± 0.276 ^b, D^	96.65 ± 0.207 ^c, D^
	6.5	100.08 ± 0.276 ^a, A^	101.91 ± 0.120 ^b, A^	101.91 ± 0.120 ^b, A^
	8.5	96.37 ± 0.276 ^a, E^	98.29 ± 0.069 ^b, E^	97.93 ± 0.069 ^c, E^

**Table 2:** Viability of *Lactiplantibacillus plantarum* pure culture and cultures supplemented with *Ascophyllum nodosum* or *Fucus vesiculosus* (0.5% *w*/*v*) during exposure to pepsin at three concentrations (0.3, 0.5, and 1.0 mg/mL). Values are expressed as log_10_ CFU/mL (mean ± standard deviation). ^a,b^ Different lowercase letters within the same row indicate significant differences (*p* < 0.05) between treatments at the same sampling time. ^A,B^ Different uppercase letters within the same column indicate significant differences (*p* < 0.05) between sampling times for the same treatment.

		Viability (log_10_ CFU/mL)		
Concentration	Time	*L. plantarum*	*L. plantarum* with *A. nodosum*	*L. plantarum* with *F. vesiculosus*
0.3 mg/mL	0	7.46 ± 0.015 ^a, A^	7.47 ± 0.017 ^a, A^	7.46 ± 0.029 ^a, A^
	1	7.37 ± 0.012 ^a, B^	7.46 ± 0.029 ^b, A^	7.42 ± 0.040 ^ab, AB^
	2	7.35 ± 0.031 ^a, B^	7.47 ± 0.021 ^b, A^	7.40 ± 0.036 ^a, AB^
	3	7.32 ± 0.020 ^a, B^	7.47 ± 0.047 ^b, A^	7.35 ± 0.031 ^ab, B^
0.5 mg/mL	0	7.43 ± 0.023 ^a, A^	7.43 ± 0.021 ^a, A^	7.43 ± 0.026 ^a, A^
	1	7.37 ± 0.017 ^a, B^	7.50 ± 0.030 ^b, B^	7.51 ± 0.006 ^b, B^
	2	7.40 ± 0.023 ^a, AB^	7.52 ± 0.026 ^b, AB^	7.53 ± 0.015 ^b, B^
	3	7.39 ± 0.020 ^a, AB^	7.47 ± 0.035 ^b, AB^	7.48 ± 0.12 ^b, B^
1 mg/mL	0	7.59 ± 0.002 ^a, A^	7.59 ± 0.002 ^a, A^	7.59 ± 0.001 ^a, A^
	1	7.48 ± 0.046 ^a, B^	7.58 ± 0.026 ^b, AB^	7.53 ± 0.023 ^b, B^
	2	7.45 ± 0.035 ^a, B^	7.56 ± 0.029 ^b, AB^	7.53 ± 0.015 ^b, B^
	3	7.45 ± 0.015 ^a, B^	7.50 ± 0.017 ^b, B^	7.50 ± 0.021 ^b, B^

**Table 3:** Viability of *Lactiplantibacillus plantarum* pure culture and cultures supplemented with *Ascophyllum nodosum* or *Fucus vesiculosus* (0.5% *w*/*v*) during exposure to trypsin at three concentrations (0.1, 0.5, and 1.0 mg/mL). Values are expressed as log_10_ CFU/mL (mean ± standard deviation). ^a,b^ Different lowercase letters within the same row indicate significant differences (*p* < 0.05) between treatments at the same sampling time. ^A,B,C^ Different uppercase letters within the same column indicate significant differences (*p* < 0.05) between sampling times for the same treatment.

		Viability (log_10_ CFU/mL)		
Concentration	Time	*L. plantarum*	*L. plantarum* with *A. nodosum*	*L. plantarum* with *F. vesiculosus*
0.1 mg/mL	0	7.54 ± 0.010 ^a, A^	7.52 ± 0.015 ^a, A^	7.51 ± 0.015 ^a, A^
	1	7.50 ± 0.012 ^a, AB^	7.61 ± 0.006 ^b, B^	7.60 ± 0.076 ^b, B^
	2	7.48 ± 0.012 ^a, AB^	7.60 ± 0.044 ^b, AB^	7.52 ± 0.046 ^ab, AB^
	3	7.43 ± 0.053 ^a, B^	7.54 ± 0.045 ^b, AB^	7.52 ± 0.055 ^b, AB^
0.5 mg/mL	0	7.65 ± 0.010 ^a, A^	7.65 ± 0.007 ^a, A^	7.64 ± 0.010 ^a, A^
	1	7.55 ± 0.031 ^a, B^	7.63 ± 0.012 ^b, AB^	7.59 ± 0.006 ^a, B^
	2	7.53 ± 0.026 ^a, B^	7.60 ± 0.021 ^b, B^	7.58 ± 0.025 ^b, B^
	3	7.52 ± 0.020 ^a, B^	7.59 ± 0.015 ^b, C^	7.58 ± 0.006 ^b, B^
1 mg/mL	0	7.59 ± 0.002 ^a, A^	7.59 ± 0.002 ^a, A^	7.59 ± 0.001 ^a, A^
	1	7.56 ± 0.006 ^a, B^	7.58 ± 0.006 ^b, AB^	7.57 ± 0.010 ^ab, B^
	2	7.53 ± 0.006 ^a, B^	7.57 ± 0.012 ^b, B^	7.57 ± 0.010 ^b, B^
	3	7.50 ± 0.010 ^a, C^	7.57 ± 0.012 ^b, B^	7.55 ± 0.012 ^b, B^

**Table 4:** Viability of *Lactiplantibacillus plantarum* pure culture and cultures supplemented with *Ascophyllum nodosum* or *Fucus vesiculosus* (0.5% *w*/*v*) during exposure to bile salts at three concentrations (0.3, 0.5, and 1.0% *w*/*v*). Values are expressed as log_10_ CFU/mL (mean ± standard deviation). ^a,b,c^ Different lowercase letters within the same row indicate significant differences (*p* < 0.05) between treatments at the same sampling time. ^A,B,C^ Different uppercase letters within the same column indicate significant differences (*p* < 0.05) between sampling times for the same treatment.

		Viability (log_10_ CFU/mL)		
**Concentration**	Time	*L. plantarum*	*L. plantarum* with *A. nodosum*	*L. plantarum* with *F. vesiculosus*
0.3% (*w*/*v*)	0	7.73 ± 0.010 ^a, A^	7.73 ± 0.006 ^a, A^	7.72 ± 0.006 ^a, A^
	1	7.67 ± 0.015 ^a, B^	7.72 ± 0.010 ^b, A^	7.70 ± 0.025 ^ab, A^
	2	7.60 ± 0.026 ^a, C^	7.69 ± 0.010 ^b, A^	7.64 ± 0.006 ^ab, B^
	3	7.55 ± 0.036 ^a, C^	7.67 ± 0.017 ^b, A^	7.63 ± 0.012 ^b, B^
0.5% (*w*/*v*)	0	7.81 ± 0.010 ^a, A^	7.83 ± 0.006 ^a, A^	7.82 ± 0.006 ^a, A^
	1	7.80 ± 0.015 ^a, AB^	7.82 ± 0.021 ^a, A^	7.82 ± 0.029 ^a, AB^
	2	7.77 ± 0.012 ^a, B^	7.80 ± 0.020 ^b, A^	7.78 ± 0.015 ^ab, B^
	3	7.69 ± 0.010 ^a, C^	7.75 ± 0.006 ^b, B^	7.74 ± 0.010 ^b, B^
1% (*w*/*v*)	0	7.97 ± 0.010 ^a, A^	7.97 ± 0.006 ^a, A^	7.96 ± 0.006 ^a, A^
	1	7.92 ± 0.007 ^a, B^	7.96 ± 0.010 ^b, AB^	7.94 ± 0.010 ^c, B^
	2	7.92 ± 0.006 ^a, B^	7.94 ± 0.002 ^b, B^	7.93 ± 0.006 ^ab, B^
	3	7.92 ± 0.012 ^a, B^	7.93 ± 0.015 ^a, B^	7.92 ± 0.010 ^a, B^

## Data Availability

The original contributions presented in this study are included in the article. Further inquiries can be directed to the corresponding author.
